# The effectiveness of low-level laser therapy for nonspecific chronic low back pain: a systematic review and meta-analysis

**DOI:** 10.1186/s13075-015-0882-0

**Published:** 2015-12-15

**Authors:** ZeYu Huang, Jun Ma, Jing Chen, Bin Shen, FuXing Pei, Virginia Byers Kraus

**Affiliations:** Department of Orthopedic Surgery, West China Hospital, West China Medical School, Sichuan University, No. 37 Wainan Guoxue Road, Chengdu, Sichuan Province People’s Republic of China; Duke Molecular Physiology Institute, Duke University School of Medicine, Duke University, PO Box 104775, Room 51-205, Carmichael Building, 300 North Duke Street, Durham, 27701-2047 NC USA; West China School of Stomatology, Sichuan University, No. 14, Third Section, Renmin Road South, Chengdu, 610041 Sichuan Province People’s Republic of China; Division of Rheumatology, Department of Medicine, Duke University School of Medicine, Durham, NC 27710 USA

**Keywords:** LLLT, Low-level laser therapy, Nonspecific chronic low back pain, NSCLBP, Pain relief

## Abstract

**Background:**

In recent decades, low-level laser therapy (LLLT) has been widely used to relieve pain caused by different musculoskeletal disorders. Though widely used, its reported therapeutic outcomes are varied and conflicting. Results similarly conflict regarding its usage in patients with nonspecific chronic low back pain (NSCLBP). This study investigated the efficacy of low-level laser therapy (LLLT) for the treatment of NSCLBP by a systematic literature search with meta-analyses on selected studies.

**Method:**

MEDLINE, EMBASE, ISI Web of Science and Cochrane Library were systematically searched from January 2000 to November 2014. Included studies were randomized controlled trials (RCTs) written in English that compared LLLT with placebo treatment in NSCLBP patients. The efficacy effect size was estimated by the weighted mean difference (WMD). Standard random-effects meta-analysis was used, and inconsistency was evaluated by the I-squared index (I^2^).

**Results:**

Of 221 studies, seven RCTs (one triple-blind, four double-blind, one single-blind, one not mentioning blinding, totaling 394 patients) met the criteria for inclusion. Based on five studies, the WMD in visual analog scale (VAS) pain outcome score after treatment was significantly lower in the LLLT group compared with placebo (WMD = -13.57 [95 % CI = -17.42, -9.72], I^2^ = 0 %). No significant treatment effect was identified for disability scores or spinal range of motion outcomes.

**Conclusions:**

Our findings indicate that LLLT is an effective method for relieving pain in NSCLBP patients. However, there is still a lack of evidence supporting its effect on function.

## Background

Low back pain (LBP) is one of the most common musculoskeletal disorders [1, 2] and the leading cause of disability worldwide [3]. It affects more than two-thirds of the population during their lifetime and one in four people seek medical help for LBP in a 6-month period [4]. Musculoskeletal disorders account for 6–8 % of total disability-adjusted life years (DALYs) and of this large total, low back pain accounts for nearly half [5]. The majority of the symptoms resolve spontaneously within 1–3 months. However, 3–10 % of patients develop chronic symptoms lasting more than 6 weeks [6]. The underlying etiology of most low back pain is currently unclear. Thus, the one term, nonspecific chronic low back pain (NSCLBP), is used to refer to this condition [7]. Annually, $91 billion in medical expenses are spent for back pain with an additional $50 billion indirect costs incurred due to loss in productivity and disability benefit payments [8, 9].

The main goal of NSCLBP therapy is rarely the complete eradication of pain. Different strategies are currently utilized including surgery and drug therapy, exercise therapy, manipulation, acupuncture, electrical treatments, and cognitive-behavioral interventions.

In recent decades, low-level laser therapy (LLLT) has been widely used to relieve pain caused by different musculoskeletal disorders [10, 11]. Though widely used, its reported therapeutic outcomes are varied and conflicting. Results similarly conflict regarding its usage in patients with NSCLBP [12, 13]. There has been a recent increase in the number of RCTs evaluating the effectiveness of LLLT in patients with NSCLBP [12–18]. Therefore, the aim of this study was to examine the totality of evidence to evaluate the effectiveness of LLLT on symptoms and function in patients with NSCLBP through a systematic review and meta-analysis.

## Methods

### Search strategy and study selection

We conducted a systematic review and meta-analysis, using the approach recommended by the PRISMA (Preferred Reporting Items for Systematic Review and Meta-Analyses) guidelines for meta-analyses of interventional studies [19]. The following bibliographic databases were searched up to 20 December 2014: Medline via PubMed from 1990, EMBASE via OVID from 1990, Web of Science from 1990 as well as the Cochrane Central Register of Controlled Trials. The search strategy was as follows: back pain OR low back pain OR backache OR lumbar adjacent pain OR chronic low back pain OR nonspecific chronic low back pain OR NSCLBP AND low-level laser therapy OR low-intensity laser therapy OR low-energy laser therapy OR LLLT OR LILT OR LELT OR infrared laser OR IR laser OR diode laser.

Two reviewers independently identified titles and abstracts relevant to LLLT for patients suffering from NSCLBP. Full texts of the published articles were analyzed and included. The reference list of the full-text articles was also reviewed. To be included in this analysis, studies had to meet the following criteria: (1) be randomized controlled trials; (2) involve patients suffering from NSCLBP; (3) compare LLLT and placebo treatment (no treatment or sham laser); (4) report pain and/or functional outcomes of patients; (5) attain a PEDro score (an 11-point scale, which is the one most often employed for physical treatments) [20] of >5 and (6) be written in English. Trials with an unbalanced additional modality between groups were excluded.

### Data extraction

Study data were extracted by two reviewers and checked for accuracy by a third reviewer including the intervention description, inclusion/exclusion criteria, baseline, demographics, and values for all outcomes at baseline and after treatment. The primary outcomes of interest were the visual analog pain (VAS) pain score and disability measured by the Oswestry disability index (ODI) [21] after treatment. The secondary outcomes of interest were change in VAS pain score (defined as the mean difference between treatment arms from baseline to follow-up) and range of motion (ROM). If the data were not presented in the study as mean and standard deviation, or were presented in a form that prevented calculation of mean and standard deviation, the original authors were contacted.

### Statistical analysis

All the primary and secondary outcomes were continuous data, permitting means and standard deviations to be used to calculate a weighted mean difference (WMD) and 95 % confidence interval (CI) in the meta-analysis. Data were presented as a forest plot. All results were checked for clinical and statistical heterogeneity. Heterogeneity was evaluated by a test for heterogeneity (I^2^ statistic): significant heterogeneity was defined as I^2^ ≤ 0.10; substantial heterogeneity was defined as I^2^ > 50 %. Data were pooled using a random-effects model. All analyses were conducted using Stata software, version 11.0 (Stata Inc., College Station, TX, USA).

## Results

### Study selection and characteristics

**Fig. 1:**
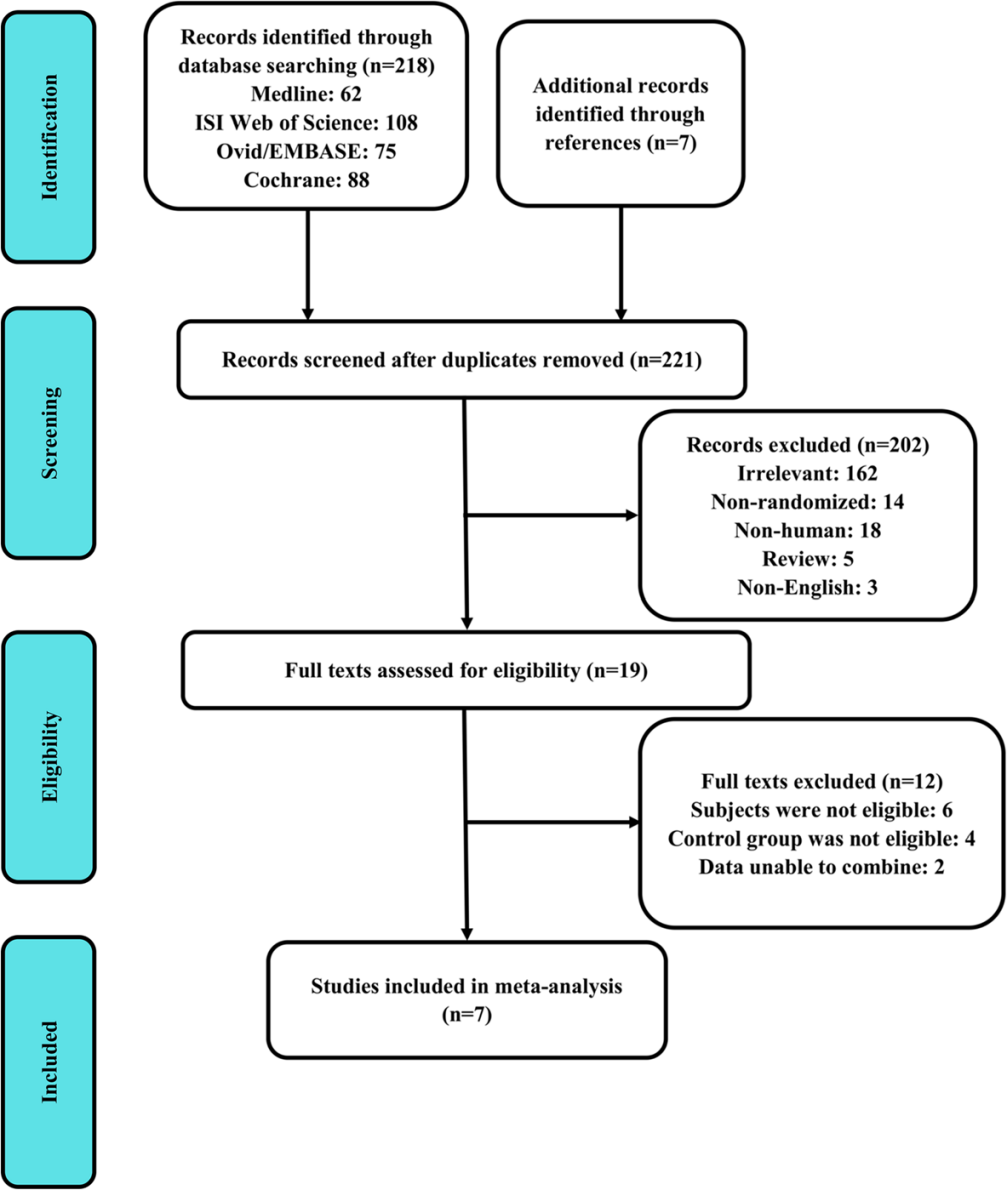
CONSORT diagram showing screening process and search results for the meta-analysis of LLLT for chronic nonspecific low back pain. *LLLT* low-level laser therapy

Figure 1 illustrates the selection process for including studies in the meta-analysis. In total, 221 potential studies were found. Based on the title and abstract content, 202 of these studies were excluded. The full texts of the remaining 19 studies were read, and a further 12 studies were excluded, resulting in seven studies [12–18] retained in the qualitative and quantitative synthesis of this review. A total of 394 patients were included: 202 patients in the LLLT group and 192 patients in the placebo group. Among the total of seven studies, three [16–18] included exercise as an additional treatment method in both study and control groups, while the other four did not. The characteristics of the included studies are listed in Table 1. Six of the seven studies achieved a high-quality PEDro score (≥7) (Table 2). Two otherwise relevant trials were excluded due to PEDro scores < =5. All outcomes with appropriately reported data were extracted and included in the meta-analysis. Outcome measures were grouped according to their construct and design (Table 3).

**Table 1:** General information on low-level laser therapy (LLLT) included in the meta-analysis

Study	Type of studies	Sample size	Age (SD) years	Gender (M/F)	Dropouts (n)	VAS pain (SD)	Intervention
Klein and Eek 1990 [13]	TB-RCT	Study group (*n* = 10)	44.1 (7.9)	2/8	0	30 (12)	LLLT vs. Placebo
		Control group (*n* = 10)	41.3 (10.7)	3/8	0	33 (11)	
Soriano and Rios 1998 [14]	DB-RCT	Study group (*n* = 38)	63.2	16/22	0	79	LLLT vs. Placebo
		Control group (*n* = 33)	64.33	18/20	0	81	
Basford et al. 1999 [15]	DB-RCT	Study group (*n* = 27)	47.8 (48.0)	18/12	3	35.2 (29.0)	LLLT vs. Placebo
		Control group (*n* = 29)	48.2 (49)	13/16	0	37.4 (36.0)	
Gur et al. 2003 [16]	SB-RCT	Study group (*n* = 25)	35.2 (10.51)	7/18	0	62 (21)	LLLT + Ex vs. Placebo + Ex
		Control group (*n* = 25)	36.4 (9.83)	8/17	0	65 (16)	
Djavid et al. 2007 [17]	DB-RCT	Study group (*n* = 19)	38 (7)	12/7	0	62 (16)	LLLT + Ex vs. Placebo + Ex
		Control group (*n* = 18)	36 (10)	15/3	0	63 (20)	
Vallone et al. 2014 [18]	RCT	Study group (*n* = 50)	68 (24-89)	43/57	0	66.4 (17.7)	LLLT + Ex vs. Placebo + Ex
		Control group (*n* = 50)			0	63.6 (15.2)	
Hsieh et al. 2014 [12]	DB-RCT	Study group (*n* = 33)	60.1 (14.2)	14/19	0	78 (24)	LLLT vs. Placebo
		Control group (*n* = 27)	58.5 (10.6)	8/19	0	79 (17)	

Mean (standard deviation) are provided above for age (years) and VAS pain (*VAS* visual analog scale)

*TB-RCT* triple blind-randomized controlled trial, *DB-RCT* double-blind randomized controlled trial, *SB-RCT* single-bind randomized controlled trial, *M* male, *F* female, *Ex* exercise

**Table 2:** Summary of methodological quality based on PEDro classification scale

Study	Item											Total
	1	2	3	4	5	6	7	8	9	10	11	
Klein and Eek 1990 [13]	✓	✓	*x*	✓	✓	✓	✓	✓	*x*	✓	✓	9
Soriano and Rios 1998 [14]	✓	✓	*x*	✓	*x*	*x*	*x*	✓	*x*	✓	✓	6
Basford et al. 1999 [15]	✓	✓	*x*	✓	✓	✓	*x*	✓	*x*	✓	✓	8
Gur et al. 2003 [16]	✓	✓	*x*	✓	✓	*x*	*x*	✓	*x*	✓	✓	7
Djavid et al. 2007 [17]	✓	✓	*x*	✓	✓	✓	*x*	✓	*x*	✓	✓	8
Vallone et al. 2014 [18]	✓	✓	✓	✓	*x*	*x*	*x*	✓	*x*	✓	✓	7
Hsieh et al. 2014 [12]	✓	✓	✓	✓	✓	*x*	✓	✓	*x*	✓	✓	9

Items: 1-eligibility criteria specified, 2-random allocation, 3-concealed allocation, 4-groups similar at baseline, 5-subject blinding, 6-therapist blinding, 7-assessor blinding, 8-less than 15 % dropouts, 9-intention-to-treat analysis, 10-between-group statistical comparisons, 11-point measures and variability data; ✓yes, *x* no

**Table 3:** Technical features of laser use in the studies included for meta-analysis

Study	Laser type	Laser model (Manufacturer)	Treatment time/Number of total sessions/Number of sessions per week	Laser continuous output	Energy density (J/cm^2^)	Energy per point (J/point per session)
Klein and Eek 1990 [13]	GaAs 904 nm	Omniprobe	20 m/12/3	20 W	1.3	1.3
Soriano and Rios 1998 [14]	GaAs 904 nm	NA	NA/10/5	40 mW	4	6*10^-6^
Basford et al. 1999 [15]	Nd: YAG 1060 nm	NA	12 m/12/3	542 mW	239.3	48.78
Gur et al. 2003 [16]	GaAs 904 nm	Class IIIb Laser Product	30 m/20/5	4.2 mW	1	1
Djavid et al. 2007 [17]	GaAlAs 810 nm	NA	20 m/12/2	50 mW	27	5.9697
Vallone et al. 2014 [18]	GaAlAs 980 nm	LEONARDO BIO	6 m/9/3	20 W	37.5	1200
Hsieh et al. 2014 [12]	GaAlAs 890 nm	Anodyne	40 m/6/3	780 mW	10.4	NA

*NA* not available

### Meta-analysis

#### Pain relief

**Fig. 2:**
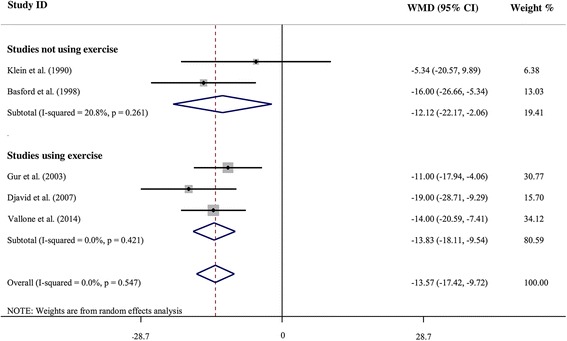
Forest plot analysis of the VAS pain score after LLLT treatment. *WMD* weighted mean difference, *LLLT* low-level laser therapy, *VAS* visual analog scale; weight % stands for the portion of the total sample contributed by each study

The mean VAS pain score after treatment was lower in the LLLT compared with the placebo group (WMD = -13.57 [95 % CI = -17.42, -9.72], I^2^ = 0 %). Subgroup analysis showed that exercise as an additional treatment did not change the results (Fig. 2). Three [12, 17, 18] of the included studies provided data on change in short-term VAS pain (follow-up minus baseline). The meta-analysis revealed significantly greater decline in VAS pain in response to LLLT compared with placebo treatment (WMD = -12.00 [95 % CI = -2.02, -21.98] I^2^ = 77.6 %) (Table 4).

**Table 4:** Meta-analyses of weighted mean differences in various continuous parameters between the LLLT and placebo groups

Outcome parameters^*^	Number of patients		Weighted mean difference (95 % CI)	*p* value	I^2^
	LLLT group (n)	Placebo group (n)			
Change in VAS pain score after treatment (from baseline to after treatment) ^12,17,18^	102	95	-12.00 [-2.02, -21.98]	0.012	77.6 %
Anterior-posterior flexion (degree)^13,17^	29	28	3.20 [-2.54, 8.93]	0.961	0 %
Anterior-posterior flexion (cm)^15,16^	35	35	-.2.84 [-5.44, 0.02]	0.480	0 %
Extension (degree)^13,17^	29	28	0.08 [-2.39, 2.54]	0.973	0 %

*LLLT* low-level laser therapy*, VAS* visual analog scale

^*^Study references provided by superscripts

#### Disability score

**Fig. 3:**
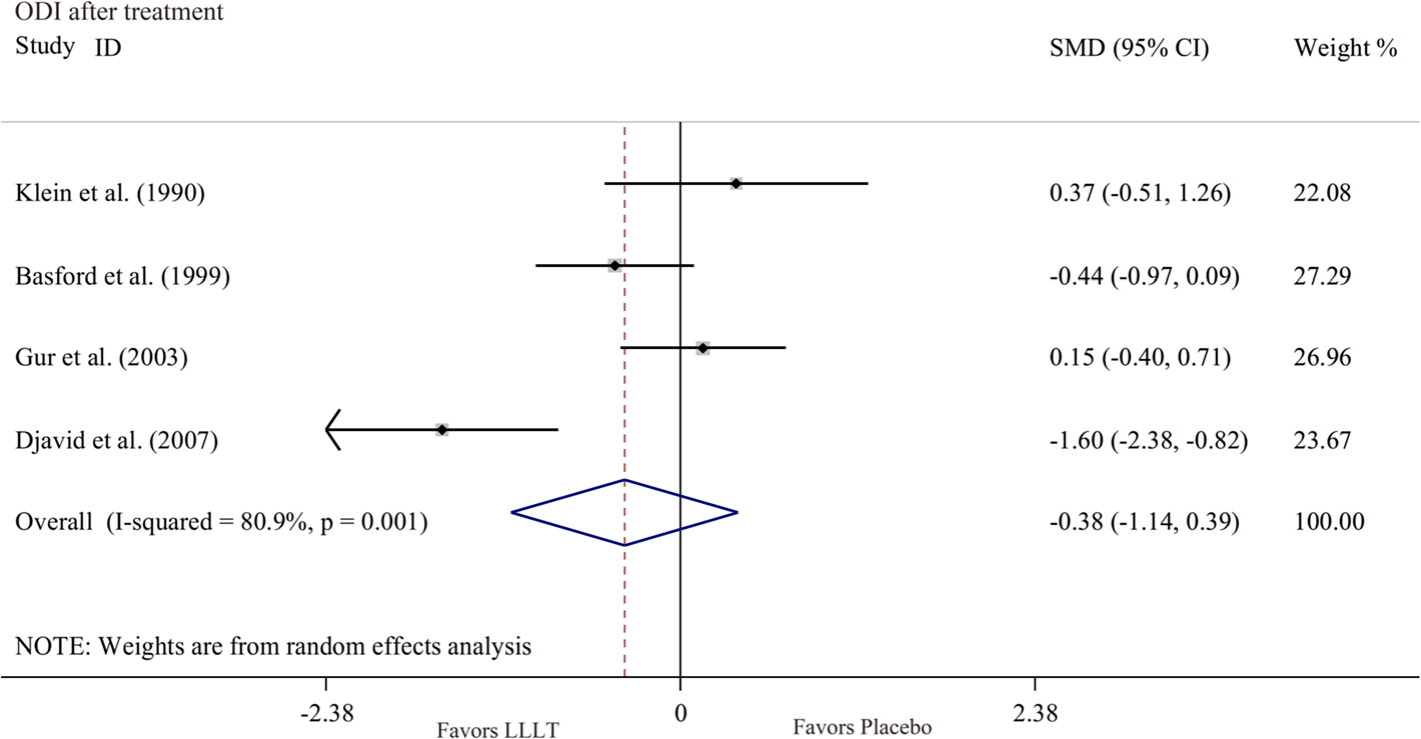
Forest plot analysis of disability outcomes after LLLT treatment measured by Oswestry disability index (ODI). *LLLT* low-level laser therapy

Disability data measured by ODI after treatment were provided by four studies [13, 15–17]. The ODI measures intensity of pain, lifting and activities such as ability to care for oneself, ability to walk, ability to sit, sexual function, ability to stand, social life, sleep quality and ability to travel. The combined results showed no significant difference between LLLT and placebo groups (WMD = -2.89 [95 % CI = -7.88, 2.29], I^2^ = 88 %) (Fig. 3).

#### ROM

Data on ROM after treatment were provided by three studies and included flexion (angle measured in degrees), anterior-posterior flexion (measured in centimeters) and extension (angle measured in degrees). The combined data in terms of these three parameters demonstrated no statistical difference between the treatment groups (Table 4).

## Discussion

NSCLBP is defined as pain of the lumbosacral area of the spine lasting more than 12 weeks. NSCLBP is a complex and multifactorial condition that may or may not have the characteristic of limiting the patient’s ROM [6]. Though various treatments options have been proposed, the management is still controversial. Most patients with NSCLBP who require medication for pain relief are likely to be middle-aged or older, and are at high risk for both adverse gastrointestinal and cardiovascular side effects [22]. Also the long-term surgical outcomes are no better than medical management [23]. Since first introduced by Mester et al. in 1968 [24], clinical application of LLLT has become more and more popular. Several experimental and clinical studies [25, 26] demonstrated its effectiveness for relief of chronic pain. Thus, many patients seek LLLT because it has no accompanying detrimental effects on systemic cardiovascular health or other adverse effects. Recently, several high-quality RCTs have emerged to assess the effectiveness of LLLT in patients with NSCLBP. We performed the current analysis, including seven RCTs with 394 patients, to gain a better understanding of the overall effect of LLLT for NSCLBP based on the higher-quality studies in a field full of seemingly conflicting results. Overall the analysis suggests that: (1) LLLT can relieve NSCLBP in a manner superior to placebo treatment; (2) LLLT is not superior to placebo treatment with respect to disability or ROM outcomes.

The mechanisms for LLLT-mediated pain relief are not fully understood. Several possible mechanisms are believed to account for the effects of LLLT, such as the following: (a) increased endogenous opioid neurotransmitter production [27]; (b) raised threshold to thermal pain and enhanced local blood circulation [28]; (c) increased oxygen consumption by accelerating the redox reaction rate of the electron respiratory chain of mitochondria [29]; (d) increased adenosine triphosphate (ATP) production at the cellular level [30]; (e) increased production of anti-inflammatory cytokines [12].

Multiple variables affect the clinical therapeutic effects of laser therapy, such as wavelength, energy density, the number of treatment sessions and their duration [31, 32]. Wavelength is also considered an essential parameter for beneficial outcomes of LLLT; it determines the ability of a laser to penetrate tissue. Wavelengths in a range of 700–1000 nm are most often used to treat deep tissues because of their superior penetration [33]. The recommended LLLT wavelengths per World Association of Laser Therapy (WALT) guidelines are 780–860 nm [34] and 904 nm [35] depending upon the condition being treated. Previous studies [36, 37] have also reported better therapeutic effects of LLLT with higher energy density, number of sessions and frequency of application. All the included studies used a wavelength within the recommended range.

It is well recognized that the effects of phototherapy are time-dependent [38]. We also observed this phenomenon as demonstrated in this meta-analysis by significant short-term but not moderate-term benefit. In contrast to pain outcomes, we did not find any significant improvement in disability or ROM due to LLLT. There might be several reasons for this. For one, the cause of NSCLBP is still unclear. Usually it is hard to determine the precise etiology of the pain. Some theories suggest that NSCLBP is linked to a reflex response of the back extensor muscles, resulting in a loss of flexion relaxation of the back muscles and a reduction of spinal flexion with secondary increased tissue strain [39]. LLLT may relieve the pain by increasing oxygen consumption and blood supply to the muscles [40]. With respect to duration, the effect is comparable to other interventions (e.g., antidepressants and traction), which are effective in the short term [41]. A primary effect on muscle may explain why we did not find any significant effect of LLLT on knee osteoarthritis (OA) pain [42], in which the sources of pain are diverse. Moreover, NSCLPB is likely a heterogeneous group of diseases, which have different etiologies but share similar symptoms. Thus, some of them might react well to LLLT while others not. Only two studies provided data on ROM. For this outcome, negative results might relate to inadequate study power that could be overcome with more high-quality investigations with ROM. Finally, like other LBP interventions, effects on pain appear to be stronger than effects on function [43].

Seven years ago, Yousefi-Nooraie et al. [44] conducted a meta-analysis of LLLT for nonspecific low back pain (NLBP); it contained seven studies of both acute and chronic NLBP [13–17, 45, 46]. The authors concluded that data were insufficient to confirm the clinical effectiveness of LLLT for NLBP. Our study is more specific, focusing on NCLBP, and is an update on this topic now demonstrating the likelihood of a beneficial effect of LLLT on low back pain. The difference in conclusions can likely be attributed to several strengths of our study. First, we focused on chronic LBP studies. The separation of chronic from acute NLBP is likely to decrease the heterogeneity. Second, the power of the current meta-analysis was substantially increased due to the inclusion of two additional studies corresponding to an appreciable increase in the numbers of subjects (sample size increased two times) over the prior meta-analysis. What is more, two low-quality studies were excluded due to PEDro scores <5 [44, 45]. Even if we included the results of theses two trials, the pooled results were still consistent with results of analyses of the trials of higher quality. Third, this meta-analysis was performed on the basis of the Cochrane Collaboration’s principle and designed to be rigorous in its search strategy. Fourth, narrow CIs around the point estimates due to the availability of higher-quality trials resulted in more precise estimates of treatment effects.

Some limitations of the current meta-analysis warrant discussion. First and foremost, we were limited by the outcome reporting of the included studies; for instance, only two studies were available for pooling to evaluate ROM. Second, because of the relatively heterogeneous treatment protocols with respect to laser parameters and laser schedules, high heterogeneity was detected in several outcomes. To overcome this, a random-effects model was chosen. Third, three of the included studies used exercise as an additional treatment in both study and control groups, while the other four did not. This could contribute to high heterogeneity in pooled results. However, the subgroup analysis showed that the use of adjunctive exercise did not affect the results. Fourth, LLLT has been also extensively used and studied in Europe and Russia; by restricting to English language studies, we could have missed some trials. Fifth, due to our relatively small number of trials, we could not assess publication bias using funnel plots or statistical tests for small sample effects.

## Conclusions

The results of our systematic review and meta-analysis have provided the best current evidence on LLLT for the treatment of NSCLBP. It suggests that LLLT is an effective method to relieve low back pain in patients who present with NSCLBP. However, there is still a lack of evidence supporting its effectiveness on functional outcomes. Further research is needed to identify the optimal LLLT parameters for achieving therapeutic efficacy, particularly for functional outcomes, and for understanding its mechanisms of action.

## Abbreviations

ATP: Adenosine triphosphate
CI: Confidence interval
DALYs: Disability-adjusted life years
LBP: Low back pain
LLLT: Low-level laser therapy
NLBP: Nonspecific low back pain
NSCLBP: Nonspecific chronic low back pain
OA: Osteoarthritis
ODI: Oswestry disability index
RCTs: Randomized controlled trials
ROM: range of motion
VAS: Visual analog scale
WALT: World Association of Laser Therapy
WMD: Weighted mean difference
